# Accounting for Biomechanical Measures from Musculoskeletal Simulation of Upright Posture Does Not Enhance the Prediction of Curve Progression in Adolescent Idiopathic Scoliosis

**DOI:** 10.3389/fbioe.2021.703144

**Published:** 2021-09-10

**Authors:** Tito Bassani, Andrea Cina, Dominika Ignasiak, Noemi Barba, Fabio Galbusera

**Affiliations:** ^1^ LABS-Laboratory of Biological Structures Mechanics, IRCCS Istituto Ortopedico Galeazzi, Milan, Italy; ^2^ Institute for Biomechanics, ETH Zurich, Zurich, Switzerland; ^3^ Department of Chemistry, Materials and Chemical Engineering “Giulio Natta”, Politecnico di Milano, Milan, Italy

**Keywords:** spine, scoliosis, progression, musculoskeletal modelling, anybody, predictive modelling

## Abstract

A major clinical challenge in adolescent idiopathic scoliosis (AIS) is the difficulty of predicting curve progression at initial presentation. The early detection of progressive curves can offer the opportunity to better target effective non-operative treatments, reducing the need for surgery and the risks of related complications. Predictive models for the detection of scoliosis progression in subjects before growth spurt have been developed. These models accounted for geometrical parameters of the global spine and local descriptors of the scoliotic curve, but neglected contributions from biomechanical measurements such as trunk muscle activation and intervertebral loading, which could provide advantageous information. The present study exploits a musculoskeletal model of the thoracolumbar spine, developed in AnyBody software and adapted and validated for the subject-specific characterization of mild scoliosis. A dataset of 100 AIS subjects with mild scoliosis and in pre-pubertal age at first examination, and recognized as stable (60) or progressive (40) after at least 6-months follow-up period was exploited. Anthropometrical data and geometrical parameters of the spine at first examination, as well as biomechanical parameters from musculoskeletal simulation replicating relaxed upright posture were accounted for as predictors of the scoliosis progression. Predicted height and weight were used for model scaling because not available in the original dataset. Robust procedure for obtaining such parameters from radiographic images was developed by exploiting a comparable dataset with real values. Six predictive modelling approaches based on different algorithms for the binary classification of stable and progressive cases were compared. The best fitting approaches were exploited to evaluate the effect of accounting for the biomechanical parameters on the prediction of scoliosis progression. The performance of two sets of predictors was compared: accounting for anthropometrical and geometrical parameters only; considering in addition the biomechanical ones. Median accuracy of the best fitting algorithms ranged from 0.76 to 0.78. No differences were found in the classification performance by including or neglecting the biomechanical parameters. Median sensitivity was 0.75, and that of specificity ranged from 0.75 to 0.83. In conclusion, accounting for biomechanical measures did not enhance the prediction of curve progression, thus not supporting a potential clinical application at this stage.

## Introduction

Adolescent idiopathic scoliosis (AIS) is a three-dimensional deformity of the spine occurring in the general population with prevalence between 2 and 3%. It begins at the time of the pubertal growth spurt and its cause is unclear (Weinstein et al., 2008; Nnadi and Fairbank, 2010). Approximately 10% of the diagnosed cases require conservative treatment and 0.1–0.3% operative correction (Negrini et al., 2018). A major clinical challenge is the difficulty of predicting curve progression at the initial presentation. The early detection of progressive curves can indeed offer the opportunity to better target effective non-operative treatments, reducing the need for surgery and the risks of related complications (Donzelli et al., 2020). The failure to accurately predict the risk of progression can lead to non-optimal treatment either by precluding timely, appropriate and efficient management or by generating unnecessary medical visits and radiographs. Moreover, uncertainty regarding curve progression and outcome can create anxiety in families and patients as well as unnecessary psychosocial stress associated with brace treatment (Weinstein et al., 2008).

Historically, curve magnitude, skeletal maturation and chronological age were considered as relevant risk factors of curve progression (Peterson and Nachemson, 1995; Kohashi et al., 1996; Lonstein and Carlson, 1984; Sanders et al., 2008; Noshchenko et al., 2015). Moreover, it was suggested that the three-dimensional shape of the scoliotic curve could be indicative of progression risk (Perdriolle and Vidal, 1981). Recently, predictive models for the early detection of the progression of scoliosis in subjects before growth spurt have been developed. Skalli et al. have proposed a severity index for classifying scoliosis as “stable” or “progressive” in subjects with mild scoliosis (Skalli et al., 2017; Vergari et al., 2019), the validation of which has been recently extended in a multicentric cohort of subjects (Vergari et al., 2021). The application requires the subjects to undergo radiographic examination by the EOS Imaging system (EOS Imaging, Paris, France), providing the simultaneous acquisition of the coronal and sagittal anatomical planes and allowing for the geometrical 3D reconstruction of the spine (Illes and Somoskeoy, 2012; Somoskeoy et al., 2012). Differently, Nault et al. evaluated mild and moderate cases and tried to predict the severity of scoliosis at full skeletal maturity (Nault et al., 2020). In both studies, the predictive models accounted for geometrical parameters describing the global spine, regional segments (scoliotic curve), or local descriptors of the curve (apex, cranial and caudal vertebrae), but neglected potential contributions from biomechanical measures.

In this regard, biomechanical parameters such as trunk muscle activation and intervertebral loading could provide additional advantageous information (Bassani et al., 2017; Schmid et al., 2020). Although not measurable *in vivo* due to the invasiveness of the procedures, such parameters can be obtained by numerical simulation based on musculoskeletal modelling approach, which allows for calculating the biomechanical loads in assigned kinematic conditions by means of inverse dynamic analysis (Dreischarf et al., 2016; Bassani and Galbusera, 2018). The present study exploits a thoracolumbar spine model with articulated ribcage, developed in AnyBody software (AnyBody Technology, Denmark) (Ignasiak et al., 2016a; Ignasiak et al., 2016b), and recently adapted and validated by our group for the subject-specific characterization of the scoliotic spine in mild severity cases (Barba et al., 2021). An existing dataset of 100 AIS subjects with mild scoliosis and in pre-pubertal age at first examination (acquired by EOS system), and recognized as stable or progressive after at least 6-months follow-up period is exploited. Anthropometrical data and geometrical parameters of the spine, as well as biomechanical parameters from musculoskeletal modelling, are accounted for as predictors of the progression of scoliosis. Six predictive modelling approaches based on different algorithms for the binary classification of stable and progressive cases are compared to find the best fitting ones. The identified models are exploited to evaluate the effect of accounting for the biomechanical parameters on the prediction of scoliosis progression. The classification performance between two sets of predictors is compared: accounting for anthropometrical and geometrical parameters, and considering in addition the biomechanical ones.

## Materials and Methods

**FIGURE 1:**
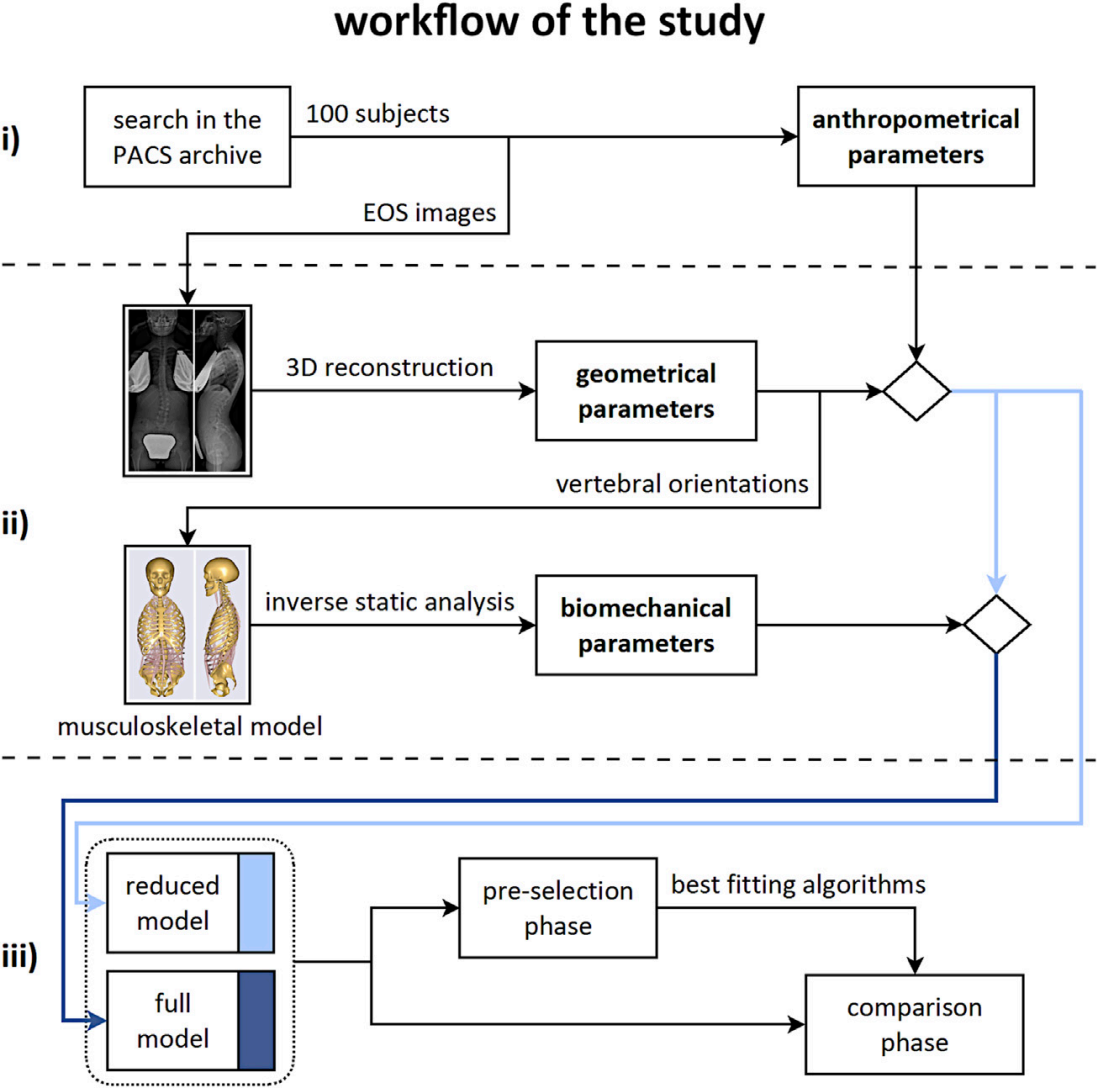
Workflow of the study. Consecutive steps from top to bottom row: i) identification of the subjects dataset and extraction of the anthropometrical parameters; ii) computation of the geometrical and biomechanical parameters; iii) evaluation of the effect of accounting for the biomechanical parameters on the prediction of the scoliosis progression.

The workflow of the study accounted for three consecutive steps (Figure 1): i) identification of the dataset of subjects and extraction of anthropometrical parameters; ii) computation of geometrical and biomechanical parameters; iii) evaluation of the effect of accounting for the biomechanical parameters on the prediction of the scoliosis progression.

### Step i)

A retrospective search of the Picture Archiving and Communication System (PACS) of the IRCCS Istituto Ortopedico Galeazzi (Milan, Italy) was performed on anonymized data acquired in the period 2014–2020. Subjects with the following criteria were included: age ranging from 10 to 18 years; at least two radiographic examinations of the spine and pelvis acquired with the EOS system. Subjects with vertebral deformities or underwent operative correction were excluded, as well as those presenting non-standard position in biplanar radiography. The Cobb angle, quantifying the severity of scoliosis in the coronal plane, and the Risser sign, determining the skeletal maturity as state of ossification and fusion of the iliac apophysis, by integer values ranging from 0 to 5 (Risser, 2010), were manually measured on the radiographic images under the supervision of an experienced spine surgeon. Subjects in the early adolescence (Risser sign ranging from 0 to 2) with mild scoliosis (Cobb angle ranging from 10 to 25) at first examination, and identified after at least 6-months follow-up period as “stable” (Risser>2, increase in Cobb angle <10 ) or “progressive” (Risser 0–2, increase in Cobb angle >10) were selected. According to that, a dataset of 100 subjects (60 stable and 40 progressive cases, respectively) was obtained. Age, sex, and Risser sign at first examination were accounted for as anthropometrical parameters.

### Step ii)

#### Geometrical Parameters

**FIGURE 2:**
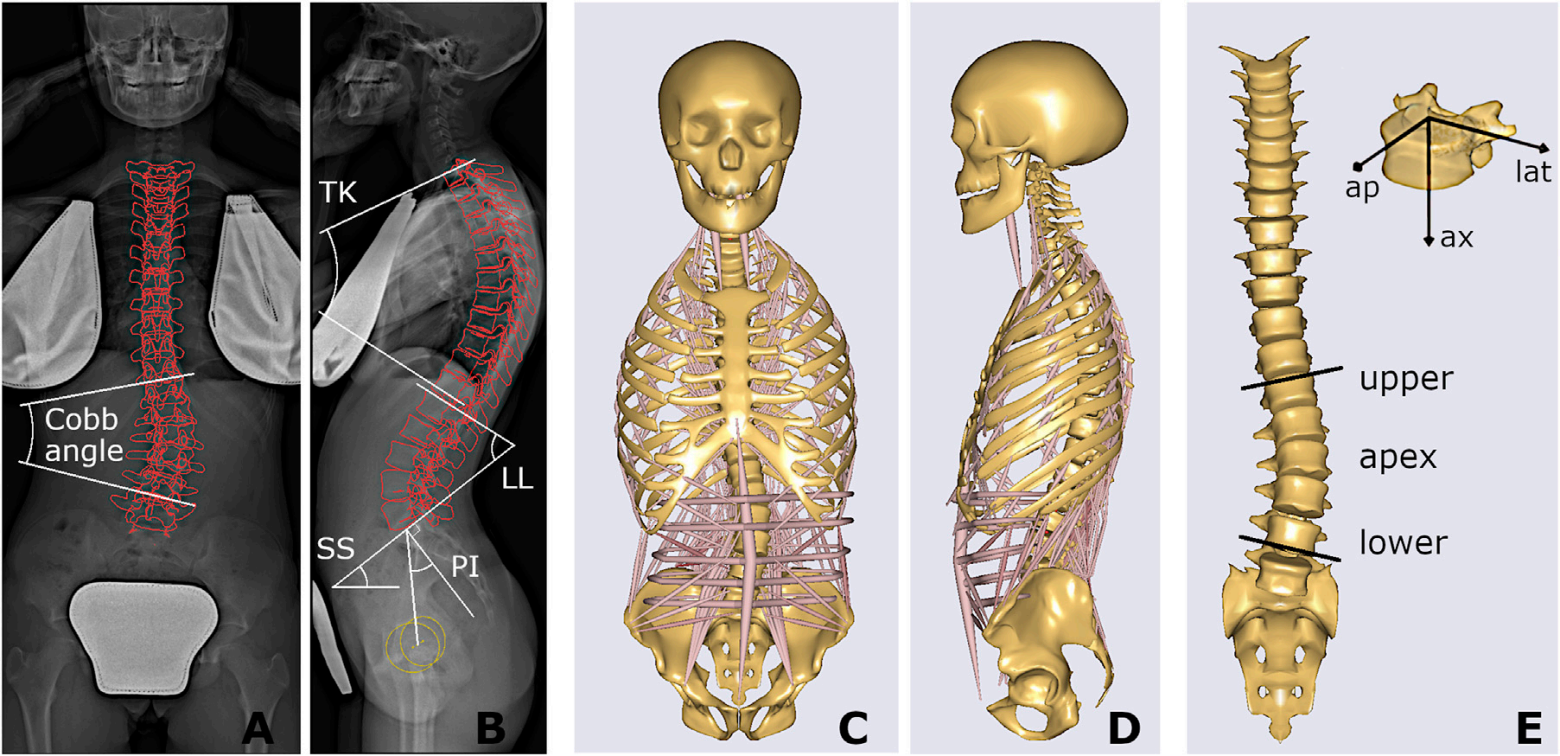
Coronal and sagittal radiographic images of one stable subject, with projection of the reconstructed vertebrae and illustration of Cobb angle, spinal sagittal alignment (TK, LL) and spinopelvic angles (SS, PI) **(A,B)**; and corresponding musculoskeletal model **(C,D)**, also presented highlighting the scoliotic curve (apex, upper and lower end levels) with muscles and ribcage not shown, and the local vertebral reference system, i.e., anteroposterior (ap), lateral (lat), and axial (ax) component **(E)**.

The radiographic images acquired at first examination (in orthostatic position with arms raised and fingertips on cheekbones) were processed by a trained operator with sterEOS software, allowing for the reconstruction of the 3D orientations of the thoracolumbar vertebrae (from T1 to L5) and the pelvis in the anatomical planes, as well as for the identification of the scoliotic curves, characterized by Cobb angle larger than 10 (Figures 2A,B) (Illes and Somoskeoy, 2012; Somoskeoy et al., 2012; Melhem et al., 2016). The following geometrical parameters were obtained: thoracic kyphosis (TK) from T1 to T12, lumbar lordosis (LL) from L1 to S1, sacral slope (SS), pelvic incidence (PI), number of scoliotic curves, Cobb angle of the most severe curve, curve sagittal angle (measuring the relative angle between the upper and lower end vertebrae in the sagittal plane), and largest vertebral axial rotation inside the curve. The type of scoliosis was determined as well according to the Lenke scheme, which classifies the deformity into six different types depending on the location and number of curves (Lenke et al., 2001). In total, nine geometrical parameters were accounted for.

#### Biomechanical Parameters

The procedure for replicating the subject-specific spinal alignment with the AnyBody musculoskeletal model (Figures 2C,D), including the rearrangement of ribs and sternum, positioning of the vertebral centers of mass, preservation of the abdominal muscle structure, setting of the trunk muscle parameters, simulation of the load of the raised arms, and muscle co-activation in maintaining the upright posture, is reported in detail in (Barba et al., 2021). In brief, the pelvis is constrained to the ground and rigidly connected to the sacrum. The spinal alignment is replicated by setting the orientation of the sacrum in the sagittal plane and the rotation of the intervertebral spherical joints from T1 to L5, according to the vertebral orientations obtained from the geometrical reconstruction. Joint moments, representing the stiffness-related contribution of passive elements such as ligaments and facet joints, are assumed as zero to replicate neutral upright position. The physiological cross-section area of the trunk muscles is scaled according to reference values acquired in adolescent subjects and depending on age (Been et al., 2018). As regards the scaling of the body model, weight and height were predicted by exploiting linear regression models taking into account anthropometrical and geometrical parameters manually measured on the radiographic images (see Appendix section), since real data were not recorded together with the images in the PACS. These models were trained by another available dataset of 85 AIS subjects with comparable age range and scoliosis severity and known weight and height data, evaluated by our group in a previous study (Bassani et al., 2019). The predicted values were exploited to scale the body model by default length-mass-fat approach. Inverse static analysis was run to calculate muscle activation and intervertebral reaction force (F) in the assigned standing posture. The activity of each muscle fascicle ranged between 0 and 1, obtained by dividing the muscle force by the maximum force generating capacity (set as the product of the cross-section area and the assumed uniform muscle stress, 90 N/cm 2). The asymmetry of erector spinae (ES) and multifidus (MF) muscle activity, between the convex and concave side of the scoliotic curve, was calculated by the normalized activity ratio (nES, and nMF) at each vertebral level inside the curve. As explained in detail in (Barba et al., 2021), this parameter is calculated by accounting for the sum of the activations of the individual fascicles crossing the respective vertebral mid-plane. It measures the (convex − concave)/(convex + concave) activity at specific vertebral level, providing values near zero in correspondence of balanced activation, and positive and negative values (ranging from 0 to ±1) in case of larger activation in the convex and concave side, respectively. As regards F, the absolute value of the intervertebral lateral shear (F_lat_), expressed in the local coordinate system of the vertebra (Figure 2E), was taken into account since expected as the most affected by lateral deviations of the spine in the coronal plane which characterize scoliosis. The following eleven biomechanical parameters were accounted for: F_lat_, nES, and nMF calculated at apex, upper and lower end levels of the scoliotic curve (Figure 2E), and nES and nMF along the whole curve, obtained by summing the contributions at all levels (from upper to lower end) in the convex and concave side. The setting steps and the simulations were run in batch process using custom routines written in MATLAB (MathWorks Inc., Natick, MA, United States), as well as the procedures for predictive modelling and statistical analysis reported in the next sections.

### Step iii)

**FIGURE 3:**
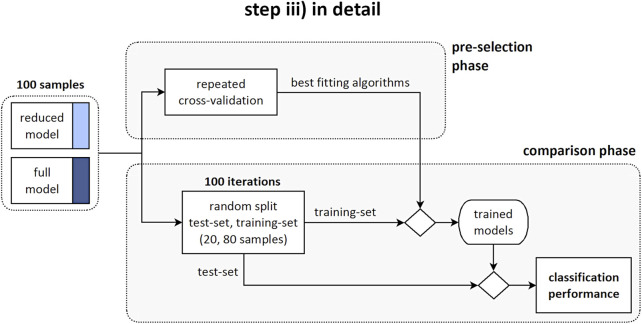
Step iii) in detail. Diagram illustrating the pre-selection phase, providing the identification of the best fitting algorithms; and the comparison phase, providing the evaluation of the classification performance between the reduced and full model.

Two sets of predictors for the binary classification of stable and progressive cases were defined. The “reduced” model accounted for 12 predictors: three anthropometrical and nine geometrical parameters (Figure 1, middle row). The “full” model accounted for the reduced set and for eleven biomechanical parameters in addition (23 predictors in total). Two consecutive processing phases were arranged: ‘pre-selection’ of the best classification approaches, and ‘comparison’ between the reduced and full model by exploiting the selected approaches (Figure 1, bottom row). Specifically, in the pre-selection phase six different algorithms for the binary classification of stable and progressive subjects were evaluated to find the best fitting approaches, both in case of reduced and full model (Figure 3). Support vector machine (SVM), predictive discriminant analysis (PDA), naive Bayes classifier (BAY), decision tree (DET), k-nearest neighbors (KNN), and ensemble method (ENS) were considered (Scholz and Wimmer, 2021; Galbusera et al., 2019; Minasny, 2009; Harper, 2005). Preliminary tuning of the hyperparameters was performed (Table1). Features selection procedures, such as principal component analysis or assessment of the correlation between parameters and binary classification, were not applied because the comparison was specifically aimed to evaluate the effect of accounting for the whole sets of available measures. Data were processed in their original format, avoiding standardization, because found as generally providing slightly larger accuracy levels (i.e., the percentage of correct predictions). Sex and Lenke type were converted into dummy variables because characterized by categorical values. The model accuracy was evaluated for each classification algorithm according to repeated cross-validation approach, by performing 10 repetitions of 4-fold cross-validation procedure (Vanwinckelen and Blockeel, 2012). This approach is appropriate for small to modestly-sized datasets and simple linear models, to reduce the noise in the estimated performance (Kuhn and Kjell 2013). In each repetition, the original dataset (100 samples) was shuffled and split into four non-overlapping folds with 25 randomly assigned samples each, preserving the original proportion of stable and progressive cases (15 (60%) and 10 (40%), respectively). Three folds at a time were used as training-set to identify the model parameters (same set for all the approaches and predictors set), and the fourth fold was exploited to compute the model accuracy. In total, the procedure provided forty values of accuracy for each evaluated model. The best fitting approaches were identified as those providing the largest average (or median) accuracy level, and were then used in the subsequent phase.In the comparison phase, the effect of accounting for the biomechanical parameters on the prediction of the scoliosis progression was evaluated by comparing the classification performance between the reduced and full model. The original dataset was randomly split into training-set and test-set (80 and 20% of total samples, respectively, preserving the original proportion of stable and progressive cases). The training-set was used to identify the models parameters (same set for each best-fitting approach and predictors set). The test-set was exploited to compute the performance of the trained models in correctly identifying the progressive and stable subjects (i.e., sensitivity and specificity of the prediction, respectively). The procedure was iterated 100 times and the average (or median) value between the reduced and full model was compared for each approach. As regards the importance of the individual predictors in determining correct classification, it is worth noting that the considered approaches are not based on modelling a direct relationship between the predictors and the binary outcome, but on finding an optimal solution by mixing information from the whole set of predictors. In general, it is thus not possible to use the estimated coefficients of the models to analyze the importance of the predictors. However, an exception is represented by DET approach. In this case, the importance of each predictor can be estimated by summing changes in the mean squared error due to splits on every predictor and dividing the sum by the number of branch nodes (Breiman, 2001). The estimation provides a positive score, which is equal to zero in case of no impact, and exhibits larger value for larger importance of the predictor.

**TABLE 1:** Hyperparameters of the classification algorithms, with tested values (range and options) and best choice (providing the largest accuracy, and used in the study) reported underlined.

	Hyperparameters
**SVM**	*box constraint*: 1–100 (10); *kernel-function*: linear, Gaussian, polynomial (2–4 order), sigmoid (with gamma: 0.0001–10, and c: 0.1–100)
**PDA**	*discriminant type*: linear, quadratic; *gamma*: 0–1 (0.6)
**BAY**	*numerical predictors distribution*: normal, kernel; *kernel options*: normal, box, epanechnikov, triangle
**DET**	max *number of splits*: 1–10 (4)*; split criterion*: Gini’s diversity index, twoing, deviance*; prune*: on, off
**KNN**	*distance metric*: euclidean, cityblock, chebychev, minkowski; *distance weight*: equal, inverse, squaredinverse; *nearest neighbours*: 1–10 (7)
**ENS**	*method*: subspace, adaBoostM1, logitBoost, gentleBoost, RUSBoost, bag*; ensemble learning cycles*: 10–100 (30)*; weak learner*: discriminant, KNN, tree

### Statistical Analysis

As regards the anthropometrical, geometrical, and biomechanical parameters, the significance of the difference between stable and progressive cases was compared by unpaired t-test (or Wilcoxon rank sum test in case of non-normal distribution) if comparing numerical values, and chi-squared test (or Fisher exact test where necessary) in case of proportions. As regards the classification performance, in the majority of cases the distribution of the accuracy values (evaluated in the pre-selection phase), and those of sensitivity and specificity (comparison phase) was found to be non-normal. According to that, the difference in the median value of accuracy among the classification algorithms was tested by Kruskal-Wallis test (separately for reduced and full model) followed by post-hoc pairwise comparisons with Tukey-Kramer approach in case of overall significance (Bassani and Galbusera, 2020). In the comparison phase, the difference in the median value of sensitivity and specificity, between the reduced and full model, was tested according to Wilcoxon rank sum test for each considered algorithm. The strength of the relation between the geometrical and biomechanical parameters was evaluated by Pearson correlation coefficient or Spearman rank in case of non-normal distribution. The significance of the coefficients in being statistically different from zero was tested according to two-tailed t test or permutation distribution test, respectively. All the tests assumed 0.05 as significance level.

## Results

### Subjects Parameters From Step i) and ii)

**FIGURE 4:**
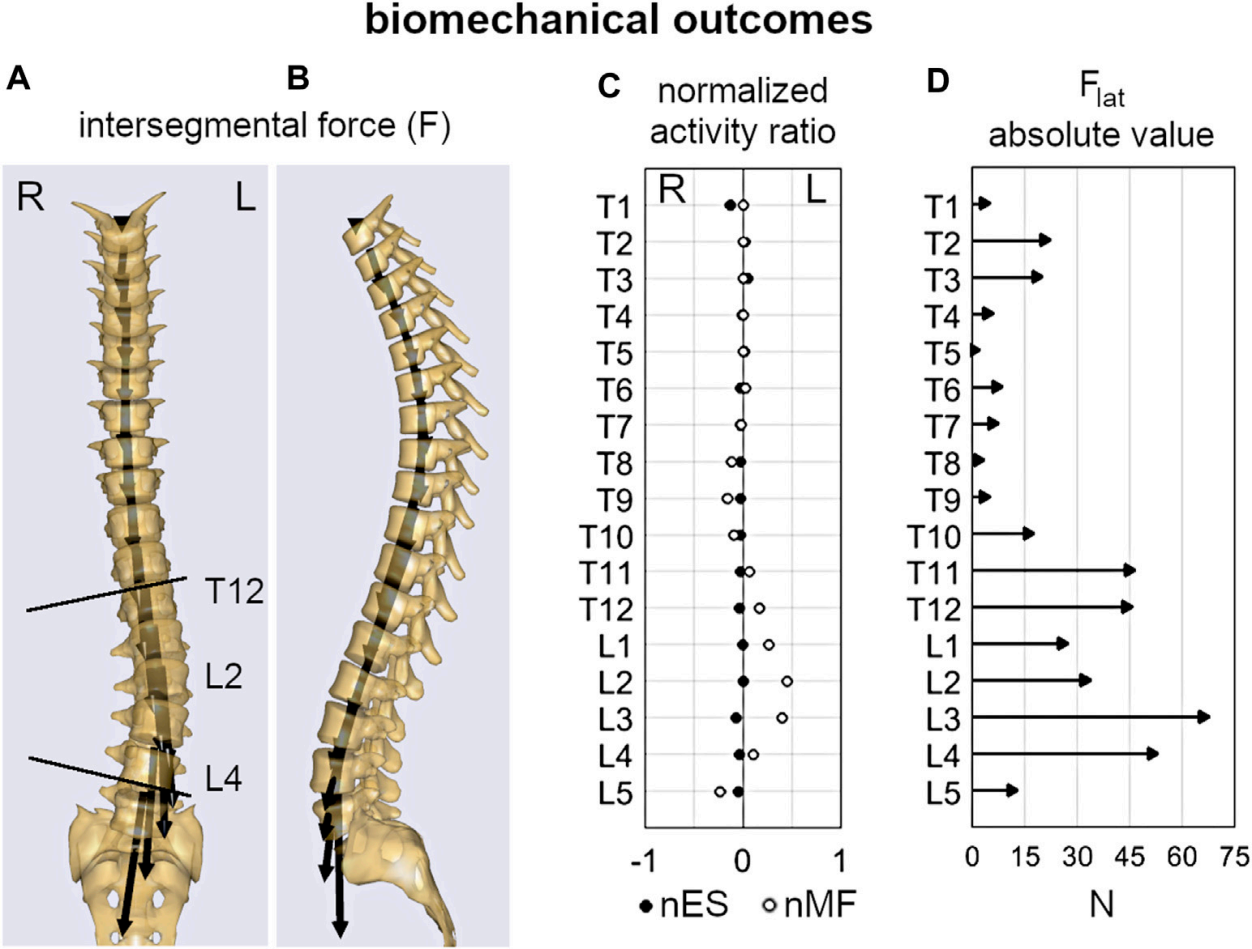
Example of the biomechanical outcomes computed by inverse static analysis for the subject depicted in Figure 2. Intersegmental force vector, F **(A,B)**; normalized activity ratio of multifidus and erector spinae (nMF and nES) muscle **(C)**; absolute value of the lateral shear, F_lat_ **(D)**.

Overall, the comparison of the average values between progressive and stable subjects pointed out slight or rather moderate differences (Table2). Age was significantly lower in the progressive cases compared to stable ones (11.5 and 13.2, *p* < 0.001), as well as Risser sign (0.2 and 1.1, *p* < 0.001). No significant differences were exhibited for sex and the other geometrical parameters, except for the curve sagittal angle (16.4 and 22.1, *p* = 0.04). As regards the biomechanical parameters, F_lat_ was found significantly lower in the progressive cases at curve apex (14.2 and 26.4, *p* < 0.01), and at upper end (35.0 and 57.8, *p* < 0.01) and lower end (46.3 and 37.9, *p* = 0.048) levels, whereas no significant differences were recognized for nES and nMF muscle activity, which exhibited slightly positive values overall (ranging from 0.02 to 0.14). An example of the distribution of the intersegmental load F, and of nES, nMF, and F_lat_, computed for a stable subject along the whole spine, is reported (Figure 4).

**TABLE 2:** values of anthropometrical and geometrical parameters, and of biomechanical parameters, expressed as mean (SD) or number of cases, for stable and progressive subjects.

Anthropometrical and geometrical parameters			Biomechanical parameters		
	Stable	Progressive		Stable	Progressive
**age** [years]	13.2 (1.1)	11.5 (1.3)^a^	**F_lat_ upper** [N]	57.8 (41.2)	35.0 (21.4) ^a^
**sex** [number of F/M subjects]	36/24	31/9	**F_lat_ apex** [N]	26.4 (22.2)	14.2 (11.6) ^a^
**Risser sign**	1.1 (0.9)	0.2 (0.5)^a^	**F_lat_ lower** [N]	46.3 (23.4)	37.9 (20.2) ^a^
**TK** [°]	44.0 (13.4)	41.4 (11.5)	**nES upper**	0.11 (0.22)	0.09 (0.19)
**LL** [°]	59.2 (9.8)	58.5 (9.9)	**nES apex**	0.12 (0.21)	0.1 (0.2)
**SS** [°]	39.6 (7.3)	39.6 (5.4)	**nES lower**	0.06 (0.19)	0.06 (0.18)
**PI** [°]	47.8 (7.8)	46.9 (7.5)	**nMF upper**	0 (0.19)	0.02 (0.11)
**number of scoliotic curves**	1.7 (0.6)	1.6 (0.5)	**nMF apex**	0.2 (0.29)	0.14 (0.22)
**Cobb angle** [°]	18.9 (6.1)	15.9 (5.1)	**nMF lower**	0.04 (0.25)	−0.03 (0.25)
**curve sagittal angle** [°]	22.1 (14.7)	16.4 (11.7)^a^	**nES curve**	0.11 (0.2)	0.08 (0.19)
**largest axial rotation** [°]	9.9 (5.6)	8.3 (5.0)	**nMF curve**	0.11 (0.22)	0.07 (0.18)
**Lenke type [cases of type 1/2/3/4/5/6]**	11/2/10/4/14/19	13/0/9/0/5/13	—	—	—

a Indicates significant difference between stable and progressive group.

### Classification Performance From Step iii)

**FIGURE 5:**
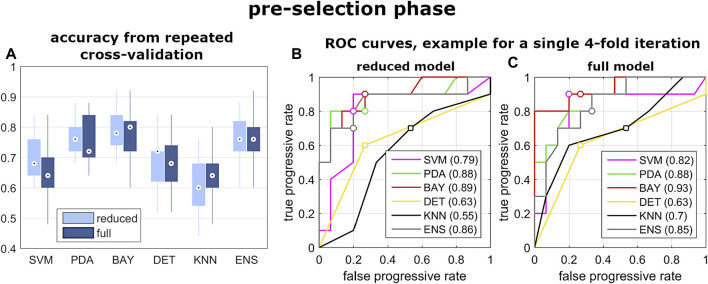
Results from the pre-selection phase of step iii). Box and whiskers plot reporting the distribution of the accuracy values obtained by the evaluated classification algorithm **(A)**; and example of ROC curves calculated by exploiting one selection of training- and test-set of the 4-folds iteration, for reduced and full model **(B,C)**.

**FIGURE 6:**
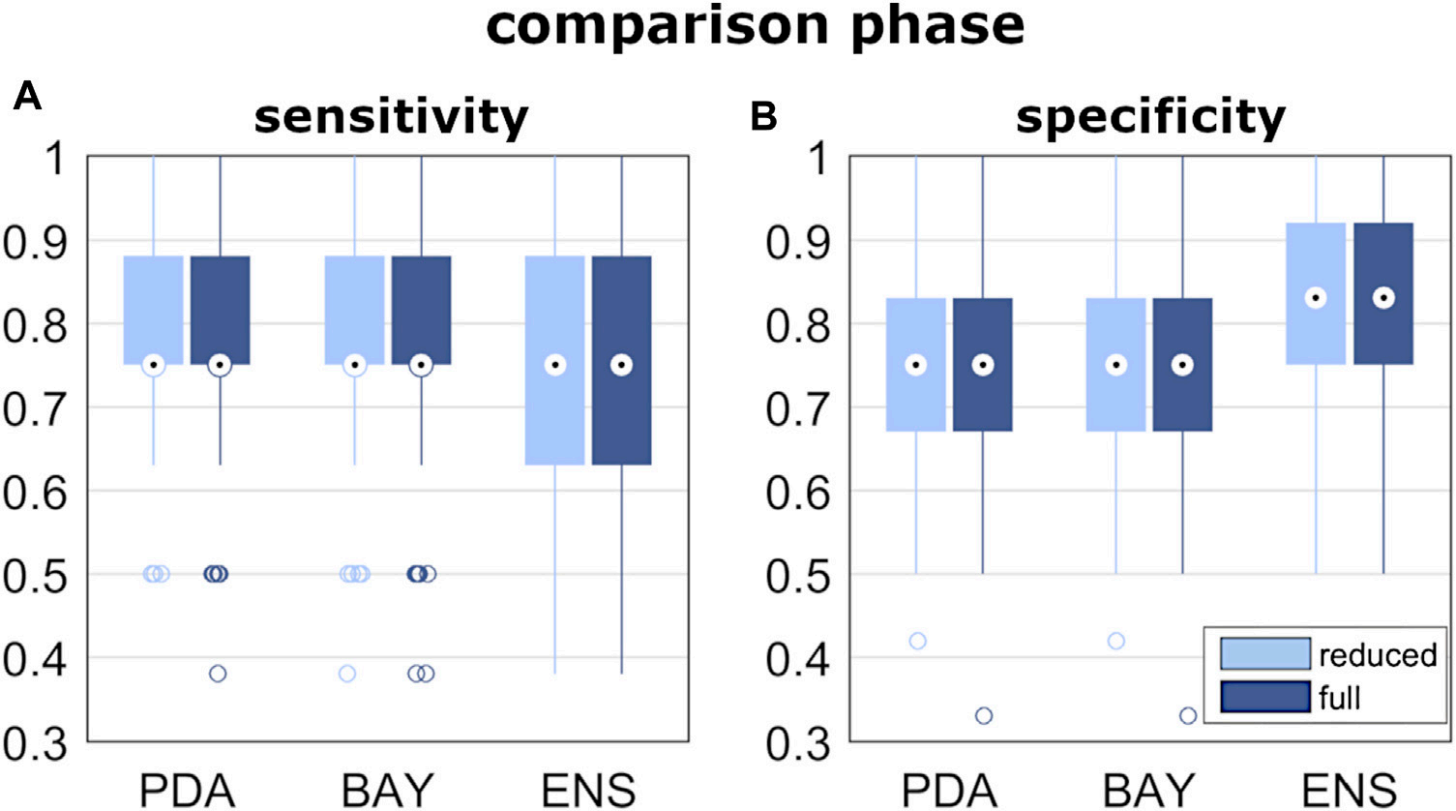
Results of classification performance, from the comparison phase of step iii). Box and whiskers plot reporting the distribution of the sensitivity and specificity values **(A,B)** obtained by the best fitting algorithms.

**FIGURE 7:**
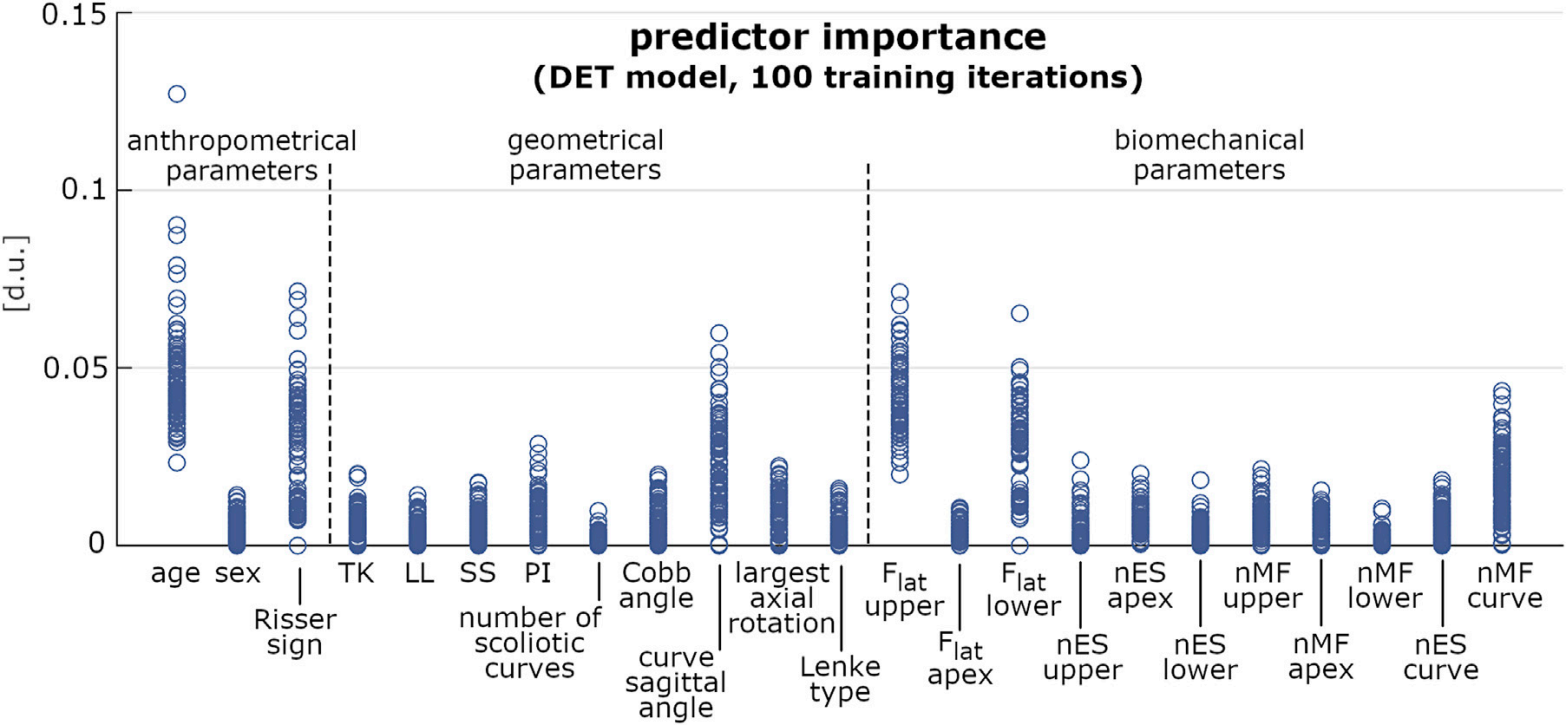
Predictor importance (expressed in dimensionless units) of the anthropometrical, geometrical and biomechanical parameters, computed for 100 iterations of model training by DET approach. Each iteration accounted for 80 samples randomly chosen in the original dataset.

In the pre-selection phase, the median accuracy of the reduced model was found significantly larger for PDA, BAY and ENS (0.76, 0.78 and 0.76, respectively) compared to SVM (0.68), DET (0.72), and KNN (0.70) (Figure 5A and Table3). Similar findings were observed with the full model: median accuracy of PDA, BAY and ENS equal to 0.72, 0.80, and 70.6, respectively, and lower values for SVM (0.64), DET (0.68), and KNN (0.64) (Figure 5A and Table3). Overall, the interquartile range (i.e., the difference between 75th and 25th percentiles) was similar among the considered conditions, with values ranging from 0.08 to 0.14. An example illustrating the ability in classifying true and false progressive cases, depicted by means of ROC curve, is reported for the reduced and full model (Figures 5B,C). The curves, obtained by processing a single selection of training- and test-set within a 4-folds split, pointed out larger values of the area under curve for PDA, BAY and ENS (ranging from 0.85 to 0.93) compared to SVM, DET, and KNN (ranging from 0.55 to 0.82). According to that, PDA, BAY and ENS algorithms were chosen as the best fitting approaches. In the comparison phase, no differences were found for sensitivity and specificity between the reduced and full model in each selected approach (Figure 6). As regards sensitivity, the same median value (0.75) was pointed out by PDA, BAY, and ENS, with larger interquartile range for ENS (0.25) compared to PDA and BAY (0.13). As regards specificity, the median value was significantly larger for ENS compared to PDA and BAY (0.83 and 0.75, *p* < 0.05), with similar interquartile range (0.16). The correlation coefficient between geometrical and biomechanical parameters was weak overall (lower than 0.3, Table4), and strong relationship (larger than 0.5) was found only between Cobb angle and F_lat_ at upper and lower end levels. As regards the importance of the predictors, that of chronological age, Risser sign, curve sagittal angle and F_lat_ at upper and lower end levels was larger compared to the other parameters (Figure 7).

**TABLE 3:** Accuracy (median and interquartile range), and statistical significance (*p*-value) of the post-hoc comparisons, among the classification algorithms (pre-selection phase, fig.5) for reduced and full model.

Reduced model						
	Accuracy	Post-hoc comparisons				
		**PDA**	**BAY**	**DET**	**KNN**	**ENS**
SVM	0.68 (0.12)	<0.01	<0.001	n.s.	<0.01	<0.01
PDA	0.76 (0.08)	—	n.s.	<0.01	<0.001	n.s.
BAY	0.78 (0.10)	—	—	<0.001	<0.001	n.s.
DET	0.72 (0.10)	—	—	—	<0.01	<0.001
KNN	0.60 (0.14)	—	—	—	—	<0.001
ENS	0.76 (0.10)	—	—	—	—	—
**full model**						
	**Accuracy**	**post-hoc comparisons**				
				**PDA**	**BAY**	**DET**	**KNN**	**ENS**
SVM	0.64 (0.10)	<0.001	<0.001	n.s.	n.s.	<0.001
PDA	0.72 (0.14)	—	n.s.	<0.05	<0.001	n.s.
BAY	0.80 (0.10)	—	—	<0.001	<0.001	n.s.
DET	0.68 (0.12)	—	—	—	n.s.	<0.001
KNN	0.64 (0.08)	—	—	—	—	<0.001
ENS	0.76 (0.08)	—	—	—	—	—

n.s. indicates not significant *p*-value.

**TABLE 4:** Correlation coefficient between geometrical and biomechanical parameters.

	F_lat_			nES			nMF			nES along curve	nMF along curve
	Upper	Apex	Lower	Upper	Apex	Lower	Upper	Apex	Lower		
**TK**	−0.07	0.06	0.07	−0.01	−0.15	−0.03	0.05	−0.25^a^	−0.26^a^	−0.1	−0.2^a^
**LL**	−0.05	−0.01	0.1	0.01	-0.03	0.1	0.03	−0.24^a^	−0.4^a^	0.01	−0.22^a^
**SS**	0.01	0	0.09	0.15	0.12	0.12	0.01	−0.06	−0.17	0.13	−0.03
**PI**	0.05	0.02	0.06	0.08	0.13	0.18	0	−0.1	−0.24^a^	0.13	−0.1
**Cobb angle**	0.63^a^	0.29^a^	0.67^a^	−.01	0.01	0.02	0.12	0.36^a^	0.11	0.02	0.3^a^
**curve sagittal angle**	−0.29^a^	−0.05	−0.12	−0.05	−0.09	−0.07	−0.12	−0.12	−0.03	−0.07	−0.1
**largest axial rotation**	0.27^a^	0.12	0.53^a^	−0.08	−0.12	−0.04	0.2	0.34^a^	0.03	−0.12	0.32^a^

a significantly different from zero.

## Discussion

The present study evaluated subjects with mild scoliosis at first examination and recognized as stable or progressive after at least 6-months follow-up period. Anthropometrical, geometrical and biomechanical parameters at first examination were extracted, and the effect of accounting for the biomechanical measures on the prediction of the scoliosis progression was assessed.

As regard the subjects’ parameters, chronological age and skeletal maturation (Risser sign) were significantly lower in the progressive cases (Table2), confirming to be relevant risk factors of curve progression (Lonstein and Carlson, 1984; Sanders et al., 2008; Noshchenko et al., 2015) and indicating that the earlier is the onset of scoliosis the higher is the probability that the deformity will increase. According to that, these factors are evaluated by clinicians as essential indicators for the choice of conservative treatment by bracing (Negrini et al., 2018). Differently, the number of curves and the type of scoliosis (Lenke type) were found as not indicative of the risk of progression, as well as the three-dimensional shape of the primary scoliotic curve. In this regard, Cobb angle, curve sagittal angle, and largest axial rotation were similar overall, although the progressive cases exhibited slightly lower values, indicating a more flat spine in the scoliotic segment. However, the sagittal and the spinopelvic alignment (TK, LL, SS, and PI) were very similar between the groups, confirming that the risk of curve progression cannot be associated a priori with changes in the geometrical parameters at the onset. As regards the biomechanical parameters, the lateral component of the intervertebral load (F_lat_) was generally lower in the progressive group at each considered level of the scoliotic curve (apex, and upper and lower end). This finding is in relation with the lower Cobb angle found for the progressive subjects compared to the stable ones (nearly significant difference, *p* = 0.06), and is in agreement with that recently observed by our group in a previous study (Barba et al., 2021). That study exploited the same musculoskeletal model to evaluate mild, moderate and severe subjects, and revealed F_lat_ as strongly correlated with scoliosis severity. In particular, the intervertebral force vector tends to be vertically oriented in the coronal plane despite the presence of deformity (see Figure 4A), whereas it is orthogonal to the vertebral upper endplate in the sagittal plane (Figure 4B). Larger deformity provides larger vertebral rotation in the coronal plane at upper and lower end levels of the scoliotic curve (Figure 2E), which results into larger contribution of the transferred load relatively to the lateral axis in the vertebral reference system (Figure 2E, upper right corner). As concerns the activation of MF and ES muscle, the slightly positive values (similar between groups) of the normalized activity ratio indicate a larger activation in the convex side of the scoliotic curve, in agreement with our previous findings (Barba et al., 2021) and with other numerical and experimental studies (Schmid et al., 2020; Cheung et al., 2005; Kwok et al., 2015). Overall, the biomechanical parameters did not provide a priori information about the risk of curve progression.

As regards the prediction of the scoliosis progression, the cross-validation analysis pointed out higher accuracy levels provided by PDA, BAY and ENS algorithm in the classification of stable and progressive cases (Figure 5), with median value ranging from 0.72 to 0.8 (Table3). This result was confirmed both in case of reduced predictors set (accounting for anthropometrical and geometrical parameters) and full set (accounting in addition for the biomechanical ones), revealing that neglecting or accounting for the biomechanical measure guaranteed very similar accuracy levels. This finding was statistically confirmed by comparing the level of sensitivity and specificity between reduced and full model (Figure 6). The median values of sensitivity (0.75 for each algorithm) and specificity (0.75 for PDA and BAY, and 0.83 for ENS) were equal for the two models. According to that, the results demonstrated that accounting for the biomechanical measures was not sufficient for enhancing the prediction of the scoliosis progression. Such unexpected outcome could be explained by hypothesizing that in the evaluated conditions (mild scoliosis and replication of static standing posture), the information obtained from the musculoskeletal simulation may reflect those provided by the geometrical reconstruction, without representing an additional advantageous contribution. As well as the geometrical parameters, the biomechanical ones provide indeed information related to the three anatomical planes, since F_lat_ is calculated in the local vertebral reference system (Figure 2), and nES and nMF are computed by summing the activation of the individual muscle fascicles, the orientation of which depends on the 3D spinal alignment and the presence of deformity. However, the weak correlation found in general between the parameters (Table4), with strong relationship only between Cobb angle and F_lat_ and depending on the orientation of the intervertebral force vector as explained above, does not support the hypothesis of redundancy between geometrical and biomechanical parameters. As regards the importance of the individual predictors in correctly classifying the scoliosis progression, chronological age and Risser confirmed to be determining (Figure 7). The curve sagittal angle also demonstrated to have an impact and this is not unexpected, since it is well recognized that a deformity in the coronal plane implicates the flattening of the corresponding spine region in the sagittal plane (Kubat and Ovadia, 2020). The lateral shear at upper and lower end levels was found important as well, and can be explained as in relation with the differences in Cobb angle discussed above. However, the Cobb angle showed lower importance, suggesting that such analysis should be taken with caution overall, and that larger datasets should be considered to better consolidate the results.

In comparison to other studies, the classification performance was moderately lower: Skalli et al. reported 0.84 and 0.89 for sensitivity and specificity (Skalli et al., 2017), and Nault at al. 0.75 and 0.94, respectively, (Nault et al., 2020). However, it is important to note that the results were obtained with different conditions of modelling strategy, number of evaluated subjects, and range of the reported results. Specifically, Skalli et al. exploited an approach based on PDA algorithm, which took into account six geometrical parameters of the primary scoliotic curve. The predictive model was trained by two control groups: non-scoliotic ones (53, stable), and cases with moderate and severe scoliosis (45, progressive). Another dataset of 65 subjects with mild scoliosis at first examination was processed by the model to determine the probability of being classified as stable or progressive, and then compared with the clinical evaluation in the follow-up. Nault at al. accounted for geometrical descriptors (more than twenty) of the global spine and scoliotic curve (Nault et al., 2014) in a dataset of 172 AIS subjects with mild and moderate scoliosis at first examination (Cobb angle ranging from 10 to 40). Their work was specifically devised to identify determinant predictors of the Cobb angle at final skeletal maturity. Descriptors found as not satisfactorily correlated with the measurement of the final Cobb angle were excluded, and an approach based on generalized linear model with backward selection was applied to find best predictors and interactions. The provided values of sensitivity and specificity were obtained as an example, by predicting those cases with final Cobb angle larger than 35. Differently from these studies, we calculated the classification performance in 100 random subsets of 20 subjects each (as described in Step iii section), and we compared the median value of sensitivity and specificity, the extent of which was found ranging from to 0.58 to 0.9 (median ± interquartile range, Table 3). As regards the evaluated predictors, we aimed to account for a list of descriptors expected as potentially related to the progression of scoliosis, avoiding similar additional parameters providing redundant information. For example, differently from that performed by Skalli et al. (2017) and Nault et al. (2020) the torsion index (the mean of the sum of the intervertebral axial rotations from lower end to apex and from apex to upper end of the scoliotic curve) was neglected in the present study. As expected, this index was found indeed significantly correlated with the largest axial rotation (0.6, *p* < 0.001), and the inclusion in the predictors set was verified as not improving the classification performance. In this respect, the index exhibited similar values in the stable and progressive group: 7.2 (4.2) and 7.1 (3.7), respectively.

The study has the following limitations. Only the relaxed upright posture was replicated, neglecting the simulation of more demanding tasks and motion activities. The development of such simulations implicates to deal with two major issues: how distributing the spine motion along the vertebral levels (i.e., the lumbar rhythm); how imposing the stiffness-related contribution of the passive elements during movements (joint moments). In this regard, reference data obtained *in vivo* or by experimental tests in AIS subjects are lacking in the literature. At this stage, we thus preferred to limit the simulation to the upright posture, although expected to provide lower spinal loads and muscle activities compared to the motion tasks (Dreischarf et al., 2016). According to that performed in previous similar works (Schmid et al., 2020; Barba et al., 2021), the evaluation of muscle activation as predictor of the scoliosis progression was limited to ES and MF. In this regard, additional groups such as quadratus lumborum, internal obliques, and latissimus dorsi could be considered as potential predictors in future developments simulating the motion of the trunk. No information about physical therapy or the prescription of bracing treatment in the period between the first examination and follow-up were available from the PACS search. The presence of that condition could represent a relevant factor since it is expected to counteract the progression of scoliosis, and neglecting such information could potentially bias the attribution of the subjects to stable or progressive group. In this regard, Skalli et al. accounted for the decision of bracing treatment in the clinical follow-up evaluation as a criterion for identifying subject as progressive (Skalli et al., 2017). Conversely, the information was neglected by Nault et al. (2020), although in a preceding study, which accounted for subjects with Cobb angle ranging from 10 to 40 at first examination, they found that bracing treatment was more present in progressive cases compared to stable ones (58 and 45% of subjects, respectively, *p* = 0.13) (Nault et al., 2014). However, bracing is usually prescribed if either of the following two conditions are met: Cobb angle >25 and significant growth left until skeletal maturity; Cobb angle <25 but rapidly progressed at the 4–6-months follow-up appointment (Negrini et al., 2018). The first condition was not met in our dataset (Cobb angle <25 at first examination as inclusion criteria). Moreover, the follow-up time (minimum 6-months as inclusion criteria) was statistically similar between the stable and progressive group (27 (13) and 25 (12) months, respectively, as mean (SD), *p* = 0.44), thus reducing the probability of a potential bias. The exploited dataset accounted for a moderate number of subjects, and larger sets should be evaluated to refine the classification models and consolidate the results. As regards the reliability of the biomechanical measures, structural peculiarities and strengths and limitations of using musculoskeletal modelling approach for the characterization of the human spine have been extensively reviewed and discussed previously (Dreischarf et al., 2016; Dao, 2016; Bassani and Galbusera, 2018). In the context of the present study, the exploited body model has been previously validated for the replication of the spinal alignment in mild scoliosis (Cobb angle <30) (Barba et al., 2021). A potential limitation is represented by the scaling of the body model by exploiting predicted values of height and weight, due to the lack of real data. In this regard, a sensitivity analysis of model outcomes based on height and weight variation was not performed. However, the predicted values are expected to be well representative of the real ones, since low prediction errors were pointed out by the corresponding predictive models (see Appendix section). Indeed, the root-mean-square error, quantifying the goodness-of-fit between real and predicted data, was found to be equal to 3.9 kg and 4.3 cm for weight and height, respectively. In conclusion, accounting for biomechanical measures obtained with musculoskeletal modelling approach, replicating the static standing posture in subjects with mild scoliosis at first examination did not enhance the prediction of the scoliosis progression. The classification performance was found very similar by including or neglecting the biomechanical parameters, although no redundancy was observed overall between the geometrical and biomechanical measures. Therefore, a potential clinical application for the early detection of the progression of the deformity is not supported at this stage. Future developments will be aimed to consolidate the results by exploiting larger datasets of subjects, to obtain relevant information from the simulation of motion tasks, and to extend the classification perspective by exploiting multinomial approaches accounting for additional conditions such as non-scoliotic subjects and severe cases.

## Figures and Tables

**FIGURE A1:**
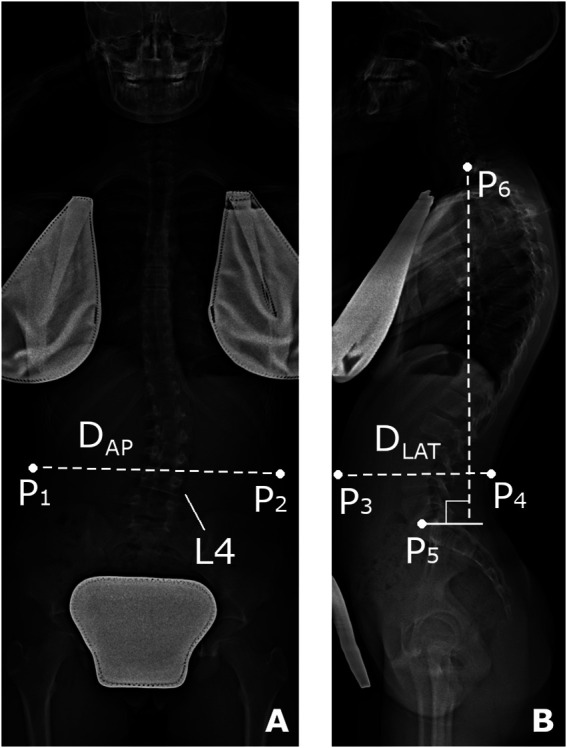
Reference points (P1-P6) handpicked on the radiographic images in the coronal **(A)** and sagittal **(B)** plane for the prediction of subject’s weight and height. Anteroposterior diameter (D_AP_) calculated as the distance between P1 and P2, lateral diameter (D_LAT_) as that between P3 and P4, and vertical distance between L5S1 and C7T1 disc center (P5 and P6, respectively).

## Data Availability

The raw data supporting the conclusions of this article will be made available by the authors, without undue reservation.

## Appendix

A procedure for predicting the subject’s weight and height from anthropometrical data and geometrical parameters measured on the radiographic images was devised, based on that proposed by O’Neill et al. (O'Neill et al., 2018), who calculated the body mass index from the cross-sectional imaging of the abdomen. A dataset of 85 AIS subjects with known weight and height, underwent radiographic examination by EOS system and evaluated by our group in a previous study was exploited (Bassani et al., 2019). The subjects were checked to be characterized by the same age range and scoliosis severity (Cobb angle <25) of those evaluated in the present study (100 AIS subjects, TableA1). The procedure accounted for the identification of six landmarks (P1-P6, Figure A1), which were handpicked in sequence on the radiographic images to provide measurements expected as strongly related to the subject’s weight and height. The points from P1 to P4 identified the maximum skin-to-skin anteroposterior and lateral diameter (D_AP_ and D_LAT_, Figure A1) in correspondence of the upper endplate center of the L4 vertebra. The effective diameter, D_E_, interpreting the diameter of the cross-sectional area at the considered level, was calculated as the square root of the product of D_AP_ and D_LAT_. Points P5 and P6 identified the center of L5S1 and C7T1 intervertebral disc, respectively, and were exploited to calculate the vertical distance between the two discs. Two independent predictive models, based on multiple linear regression, were arranged to estimate weight and height. In each model, the following set of six predictive parameters was accounted for: age, sex (converted into dummy variable), TK and LL (Figure 2B), D_E_, and discs vertical distance. The coefficients of the models were estimated by least mean squares approach (TableA2). The root-mean-square error (RMSE), quantifying the goodness-of-fit between real and predicted values, was found to be equal to 3.9 kg and 4.3 cm for weight and height, respectively. The estimated coefficients were exploited to process the six parameters as measured in the dataset of 100 AIS subjects, and to predict the corresponding values of weight and height. The distribution of the accounted parameters, as well as that of weight and height (real and predicted values, for the dataset of 85 and 100 AIS subjects, respectively), was verified to be comparable between the two datasets. In this regard, no significant differences were recognized (compared by *t*-test or Wilcoxon rank sum test) although the proportion between females and males was statistically lower in the present study (chi-square test) (Table A1). Data and image processing, and statistical analysis, were performed by custom routines written in MATLAB.

**TABLE A1:** values of the parameters, expressed as mean (SD) or number of cases, for the subjects evaluated in the present study and for the dataset with known weight and height values.

	Present study (N = 100)	Bassani et al. (2019) (N = 85)
**age** [years]	12.5 (1.5)	13.3 (1.2)
**sex** [F/M]	67/33	72/13>^a^
**Cobb angle** [°]	17.4 (5.9)	15.8 (11.4)
**TK** [°]	43.0 (12.7)	38.0 (12.3)
**LL** [°]	58.9 (9.8)	60.0 (11.0)
**D_E_** [cm]	19.9 (1.9)	20.4 (2.0)
**discs vertical distance [cm]**	38.5 (3.2)	39.1 (3.3)
**weight** [kg]	44.5 (8.0) predicted	46.6 (9.0)
**height** [cm]	159.1 (10.3) predicted	159.3 (10.6)

a indicates significant difference (*p* < 0.05) between the two study groups.

**TABLE A2:** Estimated coefficients of the linear regression models for the prediction of weight and height.

Predictor variable	Coefficient	
	Weight model	Height model
age [years]	0.8132	0.8038
sex [F/M]	−55.9553/−54.2006	45.5567/51.3015
TK [°]	0.0099	0.1886
LL [°]	0.0261	−0.0117
D_E_ [cm]	2.4337	0.5688
discs vertical distance [cm]	1.0201	2.1515

## References

[B1] Barba N. Ignasiak D. Villa T. M. T. Galbusera F. Bassani T. (2021). Assessment of Trunk Muscle Activation and Intervertebral Load in Adolescent Idiopathic Scoliosis by Musculoskeletal Modelling Approach. J. Biomech. 114, 110154. 10.1016/j.jbiomech.2020.110154 33279818

[B2] Bassani T. Galbusera F. (2018). “Musculoskeletal Modeling,” in Biomechanics of the Spine. Editors Galbusera, F. Wilke H. (Academic Press), 257–277. 10.1016/b978-0-12-812851-0.00015-x

[B3] Bassani T. Galbusera F. (2020). Statistics in Experimental Studies on the Human Spine: Theoretical Basics and Review of Applications. J. Mech. Behav. Biomed. Mater. 110, 103862. 10.1016/j.jmbbm.2020.103862 32957180

[B4] Bassani T. Ottardi C. Costa F. Brayda-Bruno M. Wilke H.-J. Galbusera F. (2017). Semiautomated 3D Spine Reconstruction from Biplanar Radiographic Images: Prediction of Intervertebral Loading in Scoliotic Subjects. Front. Bioeng. Biotechnol. 5, 1. 10.3389/fbioe.2017.00001 28164082 PMC5247473

[B5] Bassani T. Stucovitz E. Galbusera F. Brayda-Bruno M. (2019). Is Rasterstereography a Valid Noninvasive Method for the Screening of Juvenile and Adolescent Idiopathic Scoliosis? Eur. Spine J. 28, 526–535. 10.1007/s00586-018-05876-0 30617835

[B6] Been E. Shefi S. Kalichman L. F. Bailey J. Soudack M. (2018). Cross-sectional Area of Lumbar Spinal Muscles and Vertebral Endplates: a Secondary Analysis of 91 Computed Tomography Images of Children Aged 2-20. J. Anat. 233, 358–369. 10.1111/joa.12838 PMC6081509 29926903

[B7] Breiman L. (2001). Random Forests. Mach. Learn. 45, 5–32. 10.1023/a:1010933404324

[B8] Cheung J. Halbertsma J. P. K. Veldhuizen A. G. Sluiter W. J. Maurits N. M. Cool J. C. (2005). A Preliminary Study on Electromyographic Analysis of the Paraspinal Musculature in Idiopathic Scoliosis. Eur. Spine J. 14, 130–137. 10.1007/s00586-004-0780-7 15368104 PMC3476698

[B9] Dao T. T. (2016). Rigid Musculoskeletal Models of the Human Body Systems: A Review. J. Musculoskelet. Res. 19, 1630001. 10.1142/s0218957716300015

[B10] Donzelli S. Zaina F. Negrini S. (2020). Predicting Scoliosis Progression: a challenge for Researchers and Clinicians. EClinicalMedicine 18, 100244. 10.1016/j.eclinm.2019.100244 31993579 PMC6978184

[B11] Dreischarf M. Shirazi-Adl A. Arjmand N. Rohlmann A. Schmidt H. (2016). Estimation of Loads on Human Lumbar Spine: A Review of *In Vivo* and Computational Model Studies. J. Biomech. 49, 833–845. 10.1016/j.jbiomech.2015.12.038 26873281

[B12] Galbusera F. Casaroli G. Bassani T. (2019). Artificial Intelligence and Machine Learning in Spine Research. JOR Spine 2, e1044. 10.1002/jsp2.1044 31463458 PMC6686793

[B13] Harper P. R. (2005). A Review and Comparison of Classification Algorithms for Medical Decision Making. Health Policy 71, 315–331. 10.1016/j.healthpol.2004.05.002 15694499

[B14] Ignasiak D. Dendorfer S. Ferguson S. J. (2016a). Thoracolumbar Spine Model with Articulated Ribcage for the Prediction of Dynamic Spinal Loading. J. Biomech. 49, 959–966. 10.1016/j.jbiomech.2015.10.010 26684431

[B15] Ignasiak D. Ferguson S. J. Arjmand N. (2016b). A Rigid Thorax assumption Affects Model Loading Predictions at the Upper but Not Lower Lumbar Levels. J. Biomech. 49, 3074–3078. 10.1016/j.jbiomech.2016.07.006 27515441

[B16] Illés T. Somoskeöy S. (2012). The EOS Imaging System and its Uses in Daily Orthopaedic Practice. Int. Orthop. 36, 1325–1331. 10.1007/s00264-012-1512-y 22371113 PMC3385897

[B17] Kohashi Y. Oga M. Sugioka Y. (1996). A New Method Using Top Views of the Spine to Predict the Progression of Curves in Idiopathic Scoliosis during Growth. Spine 21, 212–217. 10.1097/00007632-199601150-00010 8720406

[B18] Kubat O. Ovadia D. (2020). Frontal and Sagittal Imbalance in Patients with Adolescent Idiopathic Deformity. Ann. Transl. Med. 8, 29. 10.21037/atm.2019.10.49 32055620 PMC6995921

[B19] Kuhn M. Kjell J. (2013). Applied Predictive Modeling. New York: Springer.

[B20] Kwok G. Yip J. Cheung M. C. Yick K. L. (2015). Evaluation of Myoelectric Activity of Paraspinal Muscles in Adolescents with Idiopathic Scoliosis during Habitual Standing and Sitting. Biomed. Res. Int. 2015, 958450. 10.1155/2015/958450 26583151 PMC4637075

[B21] Lenke L. G. Betz R. R. Harms J. Bridwell K. H. Clements D. H. Lowe T. G. (2001). Adolescent Idiopathic Scoliosis. J. Bone Jt. Surg. Am. 83, 1169–1181. 10.2106/00004623-200108000-00006 11507125

[B22] Lonstein J. E. Carlson J. M. (1984). The Prediction of Curve Progression in Untreated Idiopathic Scoliosis during Growth. J. Bone Jt. Surg. 66, 1061–1071. 10.2106/00004623-198466070-00013 6480635

[B23] Melhem E. Assi A. El Rachkidi R. Ghanem I. (2016). EOS Biplanar X-ray Imaging: Concept, Developments, Benefits, and Limitations. J. Child. Orthop. 10, 1–14. 10.1007/s11832-016-0713-0 26883033 PMC4763151

[B24] Minasny B. (2009). The Elements of Statistical Learning. Springer Series in Statistics, 745.

[B25] Nault M.-L. Beauséjour M. Roy-Beaudry M. Mac-Thiong J.-M. de Guise J. Labelle H. (2020). A Predictive Model of Progression for Adolescent Idiopathic Scoliosis Based on 3D Spine Parameters at First Visit. Spine (Phila Pa. 1976) 45, 605–611. 10.1097/brs.0000000000003316 31703055

[B26] Nault M.-L. Mac-Thiong J.-M. Roy-Beaudry M. Turgeon I. Deguise J. Labelle H. (2014). Three-Dimensional Spinal Morphology Can Differentiate between Progressive and Nonprogressive Patients with Adolescent Idiopathic Scoliosis at the Initial Presentation. Spine. (1976) 39, E601–E606. 10.1097/brs.0000000000000284 PMC4047302 24776699

[B27] Negrini S. Donzelli S. Aulisa A. G. Czaprowski D. Schreiber S. de Mauroy J. C. (2018). 2016 SOSORT Guidelines: Orthopaedic and Rehabilitation Treatment of Idiopathic Scoliosis during Growth. Scoliosis 13, 3. 10.1186/s13013-017-0145-8 PMC5795289 29435499

[B28] Nnadi C. Fairbank J. (2010). Scoliosis: a Review. Paediatr. Child. Health 20, 215–220. 10.1016/j.paed.2009.11.009

[B29] Noshchenko A. Hoffecker L. Lindley E. M. Burger E. L. Cain C. M. Patel V. V. (2015). Predictors of Spine Deformity Progression in Adolescent Idiopathic Scoliosis: A Systematic Review with Meta-Analysis. World J. Orthop. 6, 537–558. 10.5312/wjo.v6.i7.537 26301183 PMC4539477

[B30] O'Neill S. Kavanagh R. G. Carey B. W. Moore N. Maher M. O'Connor O. J. (2018). Using Body Mass index to Estimate Individualised Patient Radiation Dose in Abdominal Computed Tomography. Eur. Radiol. Exp. 2, 38. 10.1186/s41747-018-0070-5 30483977 PMC6258803

[B31] Perdriolle R. Vidal J. (1981). A Study of Scoliotic Curve. The Importance of Extension and Vertebral Rotation (Author's Transl). Rev. Chir. Orthop. Reparatrice Appar. Mot. 67, 25–34. 6453393

[B32] Peterson L. E. Nachemson A. L. (1995). Prediction of Progression of the Curve in Girls Who Have Adolescent Idiopathic Scoliosis of Moderate Severity. Logistic Regression Analysis Based on Data from the Brace Study of the Scoliosis Research Society. J. Bone Jt. Surg. 77, 823–827. 10.2106/00004623-199506000-00002 7782354

[B33] Risser J. C. (2010). The Classic: The Iliac Apophysis: an Invaluable Sign in the Management of Scoliosis. 1958. Clin. Orthop. Relat. Res. 468, 643–653. 10.1007/s11999-009-1096-z 19763720 PMC2816746

[B34] Sanders J. O. Khoury J. G. Kishan S. Browne R. H. Mooney J. F. 3rd Arnold K. D. (2008). Predicting Scoliosis Progression from Skeletal Maturity: a Simplified Classification during Adolescence. J. Bone Jt. Surgery Am. 90, 540–553. 10.2106/jbjs.g.00004 18310704

[B35] Schmid S. Burkhart K. A. Allaire B. T. Grindle D. Bassani T. Galbusera F. (2020). Spinal Compressive Forces in Adolescent Idiopathic Scoliosis with and without Carrying Loads: A Musculoskeletal Modeling Study. Front. Bioeng. Biotechnol. 8, 159. 10.3389/fbioe.2020.00159 32195239 PMC7062648

[B36] Scholz M. Wimmer T. (2021). A Comparison of Classification Methods across Different Data Complexity Scenarios and Datasets. Expert Syst. Appl. 168, 114217. 10.1016/j.eswa.2020.114217

[B37] Skalli W. Vergari C. Ebermeyer E. Courtois I. Drevelle X. Kohler R. (2017). Early Detection of Progressive Adolescent Idiopathic Scoliosis. Spine (Phila Pa. 1976) 42, 823–830. 10.1097/brs.0000000000001961 27779608

[B38] Somoskeöy S. Tunyogi-Csapó M. Bogyó C. Illés T. (2012). Accuracy and Reliability of Coronal and Sagittal Spinal Curvature Data Based on Patient-specific Three-Dimensional Models Created by the EOS 2D/3D Imaging System. Spine J. 12, 1052–1059. 10.1016/j.spinee.2012.10.002 23102842

[B39] Vanwinckelen G. Blockeel H. (2012). “On Estimating Model Accuracy with Repeated Cross-Validation,” in BeneLearn 2012: Proceedings of the 21st Belgian-Dutch Conference on Machine Learning, Ghent, Belgium, May 24–25, 2012, 39–44.

[B40] Vergari C. Gajny L. Courtois I. Ebermeyer E. Abelin-Genevois K. Kim Y. (2019). Quasi-automatic Early Detection of Progressive Idiopathic Scoliosis from Biplanar Radiography: a Preliminary Validation. Eur. Spine J. 28, 1970–1976. 10.1007/s00586-019-05998-z 31076919

[B41] Vergari C. Skalli W. Abelin-Genevois K. Bernard J. C. Hu Z. Cheng J. C. Y. (2021). Effect of Curve Location on the Severity index for Adolescent Idiopathic Scoliosis: a Longitudinal Cohort Study. Eur. Radiol. In press. 10.1007/s00330-021-07944-4 33884474

[B42] Weinstein S. L. Dolan L. A. Cheng J. C. Danielsson A. Morcuende J. A. (2008). Adolescent Idiopathic Scoliosis. Lancet 371, 1527–1537. 10.1016/s0140-6736(08)60658-3 18456103

